# The impact of cow's milk-mediated mTORC1-signaling in the initiation and progression of prostate cancer

**DOI:** 10.1186/1743-7075-9-74

**Published:** 2012-08-14

**Authors:** Bodo C Melnik, Swen Malte John, Pedro Carrera-Bastos, Loren Cordain

**Affiliations:** 1 Department of Dermatology, Environmental Medicine and Health Theory, University of Osnabrück, Sedanstrasse 115, Osnabrück, D-49090, Germany; 2 Center for Primary Health Care Research, Lund University, Lund, Sweden; 3 Department of Health and Exercise Science, Colorado State University, Fort Collins, CO, 80523, USA

**Keywords:** Cancer prevention, Dairy, Estrogens, IGF-1, Insulin, Leucine, Metformin, Milk signaling, Morphogenesis, mTORC1, Prostate cancer

## Abstract

Prostate cancer (PCa) is dependent on androgen receptor signaling and aberrations of the PI3K-Akt-mTORC1 pathway mediating excessive and sustained growth signaling. The nutrient-sensitive kinase mTORC1 is upregulated in nearly 100% of advanced human PCas. Oncogenic mTORC1 signaling activates key subsets of mRNAs that cooperate in distinct steps of PCa initiation and progression. Epidemiological evidence points to increased dairy protein consumption as a major dietary risk factor for the development of PCa. mTORC1 is a master regulator of protein synthesis, lipid synthesis and autophagy pathways that couple nutrient sensing to cell growth and cancer. This review provides evidence that PCa initiation and progression are promoted by cow´s milk, but not human milk, stimulation of mTORC1 signaling. Mammalian milk is presented as an endocrine signaling system, which activates mTORC1, promotes cell growth and proliferation and suppresses autophagy. Naturally, milk-mediated mTORC1 signaling is restricted only to the postnatal growth phase of mammals. However, persistent consumption of cow´s milk proteins in humans provide highly insulinotropic branched-chain amino acids (BCAAs) provided by milk´s fast hydrolysable whey proteins, which elevate postprandial plasma insulin levels, and increase hepatic IGF-1 plasma concentrations by casein-derived amino acids. BCAAs, insulin and IGF-1 are pivotal activating signals of mTORC1. Increased cow´s milk protein-mediated mTORC1 signaling along with constant exposure to commercial cow´s milk estrogens derived from pregnant cows may explain the observed association between high dairy consumption and increased risk of PCa in Westernized societies. As well-balanced mTORC1-signaling plays an important role in appropriate prostate morphogenesis and differentiation, exaggerated mTORC1-signaling by high cow´s milk consumption predominantly during critical growth phases of prostate development and differentiation may exert long-term adverse effects on prostate health. Attenuation of mTORC1 signaling by contemporary Paleolithic diets and restriction of dairy protein intake, especially during mTORC1-dependent phases of prostate development and differentiation, may offer protection from the most common dairy-promoted cancer in men of Western societies.

## Introduction

Prostate cancer (PCa) is the most commonly diagnosed malignancy in males living in highly developed and industrialized countries of Europe and North America. In these Western countries high PCa incidence rates between 80 and 100 per 100,000 per year are observed, while incidence rates of PCa in western, southeastern and eastern Asia and eastern and northern Africa range between 10 and 20 per 100,000 per year [1]. There were an estimated 217,730 new PCa diagnoses and 32,050 deaths in the United States during 2010, which makes PCa the second leading cause of cancer death in men. Several lines of evidence confirmed that Western diet is related to PCa risk and outcome [2-5]. In addition, per capita consumption of milk and dairy products correlates positively with both PCa incidence and mortality [6,7].

### Epidemiological association between dairy protein consumption and prostate cancer

**Figure 1:**
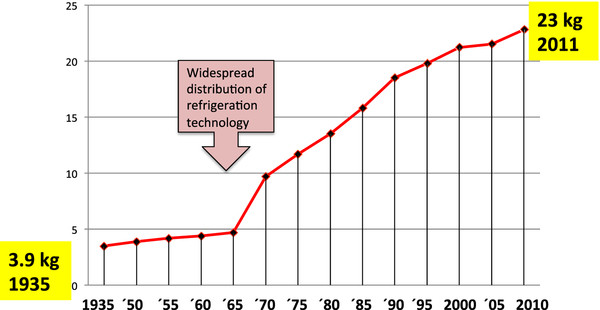
** Annual increase of per capita cheese consumption in Germany.** Cheese is a rich source of the mTORC1-activating amino acid leucine. Cheese consumption steadily increased in industrialized countries like Germany.

Diets of wealthy well-developed countries are characterized by high dairy protein and meat consumption. In Japan the mortality of PCa increased 25-fold linearly after World War 2 associated with an increase in the intake of milk (20-fold), meat (9-fold) and eggs (7-fold), respectively [8]. *The Health Professionals Study* demonstrated a strong association between calcium intake and prostate cancer risk [9]. Dairy proteins are a significant dietary source of calcium. In Western diets, dairy protein-associated calcium is predominantly provided by high and increasing consumption of cheese exemplified by the per capita cheese consumption in Germany from 1935 to 2011 (Figure 1). Whereas, Giovannucci *et al.*[9] suggested that high calcium intake might increase prostate carcinogenesis by lowering serum concentrations of 1,25-dihydroxyvitamin D [1,25(OH)_2_D], they could not exclude a role of “additional cancer-promoting factors in the nonfat component” of dairy products. *The European Prospective Investigation into Cancer and Nutrition* examined animal food, protein and calcium consumption and the risk of PCa in 142,251 men during an 8.7-year prospective study period and confirmed a strong association between high intake of dairy protein and increased risk of PCa [10]. An increase of 35 g/day in consumption of dairy protein was associated with an increase in the risk of PCa of 32% [10]. 

Remarkably, calcium from dairy products was positively associated with PCa risk, but not calcium from other foods [10]. This observation questions the role of dairy-derived calcium and points to a more critical role of the *milk protein fraction* itself. In fact, Ahn *et al.*[11] found no association between calcium intake and serum concentrations of 25-hydroxy-vitamin D [25(OH)D] and 1,25(OH)_2_D. Moreover, the calcium-vitamin-D hypothesis has been challenged at the level of cell biology by Heaney [12]. Thus, recent lines of evidence do not support the formerly suggested “high calcium intake/low vitamin D hypothesis” of dairy-mediated prostate tumorigenesis [9]. In this paper, evidence will be provided that the *intrinsic signaling capability of the dairy protein* itself is the most critical nutritional factor linking milk and dairy products to the pathogenesis of PCa.

Several ecological, cohort and case control studies conducted in various countries [6,8-11,13-21] have provided evidence for the association between increased milk and dairy intake and increased risk of PCa, which has been confirmed by meta-analyses and systematic reviews [22-25]. However, two studies, the prospective cohort study of Rodriguez *et al.*[26] and the meta-analysis of Huncharek *et al.*[27], did not find an association between dairy product intake and increased risk of PCa. In contrast, Raimondi *et al.*[21] recently reported a two-fold increased risk of PCa associated with high intakes of dairy products. Ganmaa *et al.*[6] have analyzed the incidence and mortality rates of PCa in 42 countries and identified “milk + cheese” as a major risk constellation contributing to the mortality from PCa. Notably, populations with low dairy protein intake like the Inuit and Alaska native men exhibit an extremely low incidence and mortality rate of PCa [28,29]. It is alarming, that daily milk consumption in adolescence has recently been associated with a 3.2-fold risk of advanced PCa in adulthood [30]. Notably, milk and dairy consumption has been linked to an increased incidence of acne in adolescence [31-35]. Severe acne in adolescence has been related to an increased risk of PCa in adulthood [36]. The association between PCa and acne might already point to overstimulated mTORC1-signaling of the androgen-dependent sebaceous gland and prostate gland during puberty as the underlying common cause of aberrant signal transduction [37].

As the majority of controlled studies have shown a dose-related association between dairy protein intake and increased risk of PCa, the underlying mechanism of dairy-induced PCa has not yet been identified. The calcium hypothesis [9,38], however, does not explain the cancer promoting effects of dairy protein intake as non-dairy-derived calcium showed no relation to an increased risk of PCa [10]. Neither intake of calcium from dairy products nor calcium supplements were associated with PCa risk in a prospective study of Koh *et al.*[39]. Neither high calcium nor high phosphate intake of dairy products have been shown to change intracellular concentrations of 1,25(OH)_2_D in PCa cells. Intracellular 1,25(OH)_2_D concentrations are primarily regulated by intracellular synthesis and not by 1,25(OH)_2_D uptake from the circulation [12]. Thus, neither the calcium hypothesis [38] nor the phosphorus hypothesis of PCa [40] provide reasonable tumorigenic mechanisms compatible with recent insights into the molecular biology of vitamin D, which could explain the proliferative cellular effects of dairy intake, thus calling both hypotheses of PCa into question. It is the major purpose of this paper to provide evidence that dairy protein-derived amino acids provide the cancer-promoting effect of dairy by introducing mammalian milk as a fundamental growth-promoting mTORC1-signaling system of mammalian evolution.

### Evidence for cows´milk growth-stimulatory effects on prostate cancer

Organic cows' milk, digested *in vitro*, stimulated the growth of LNCaP prostate cancer cells in each of 14 separate experiments, producing an average increase in growth rate of over 30% [41]. Remarkably, isolated addition of digested purified casein had a less stimulatory effect on LNCaP cell proliferation than whole cow´s milk [41]. This observation implies that the hormonal compounds of commercial cows milk like estrogens and IGF-1 are not the exclusive stimuli of milk-induced PCa cell growth, but rather points to the role of amino acids derived from hydrolysed milk proteins. This *in vitro* evidence fits well with recent epidemiological data from a cohort of 3,918 men diagnosed with apparently localized PCa. In this cohort, high versus low intakes of whole milk substantially increased the risk of PCa progression [42]. To understand the biological impact of milk in prostate tumorigenesis, the signal transduction pathways driven by mammalian milk, an mTORC1-signaling system naturally confined to the neonatal growth period, must be examined in greater detail.

### mTORC1: the convergence point of nutrient-derived and milk-mediated signaling

Milk signaling is integrated and mediated by the nutrient-sensitive kinase mTORC1 (*mammalian target of rapamycin complex 1*). mTORC1 links amino acid, growth factor, and energy availability to prostate epithelial cell growth, proliferation, motility, autophagy, morphogenesis and tumorigenesis. The mTORC1 signaling pathway has become a major focus of human cancer research [43]. It is thus of utmost importance to understand the nutrient-mediated signaling pathways regulating mTORC1, the central hub controlling cell signaling, cell growth and cell proliferation.

mTORC1 signaling stimulates gene transcription, translation, ribosome biogenesis, protein synthesis, insulin synthesis, cell growth, cell proliferation, lipid synthesis but suppresses mechanisms of autophagy [44-49]. mTOR is a multi-domain protein of approximately 300 kDa exhibiting a protein kinase domain at its C-terminus related to phosphoinositol-3-kinases (PI3Ks). In mammalian cells two functionally different mTOR complexes exist: *mTORC1* and *mTORC2*. Among other functional proteins, mTORC1 contains the important partner protein *Raptor*, which interacts with substrates for mTORC1-mediated phosphorylation like p70 S6 kinase 1 (S6K1). mTORC1 controls the impact of nutrient-derived signals on the G_1_/S transition and G_2_/M progression of the cell cycle [46]. In contrast to mTORC2, which contains the partner protein *Rictor*, only mTORC1 plays a special role in sensing cellular nutrients, amino acids, and energy (ATP) levels, which are important stimuli for cell growth and proliferation. Liver kinase B1 (LKB1) and AMP-activated protein kinase (AMPK) are further critical regulators of mTORC1 [50]. Most functions of mTORC1 are inhibited by rapamycin, a triene macrolide antibiotic synthesized by *Streptomyces hygroscopicus*[51].

### Pivotal role of the essential amino acid leucine for mTORC1 activation

**Figure 2:**
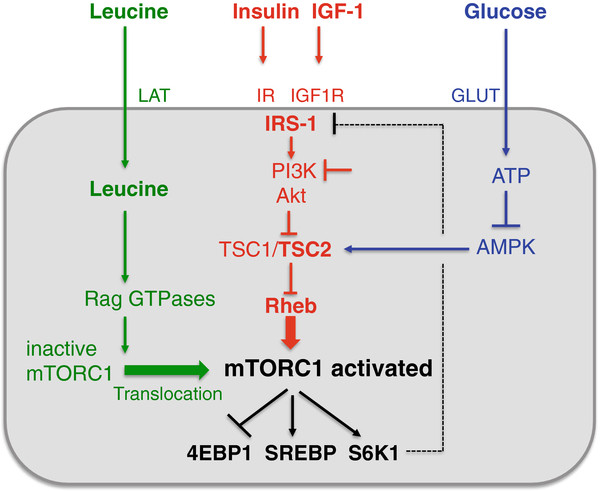
** Synopsis of major pathways activating mTORC1.** mTORC1 is activated by leucine, insulin, IGF-1 and glucose. The activating signals of the growth factors insulin and IGF-1 and the energy source glucose are mediated by attenuating the inhibitory function of TSC2 towards Rheb. The key amino acid leucine activates mTORC1 independent of the TSC2 pathway. Leucine stimulates Rag GTPase-mediated translocation of inactive mTORC1 to endosome/lysosome membranes enriched in activated Rheb. See list of abbreviations.

mTORC1 activation is critically dependent on the availability of sufficient amounts of amino acids, especially of the branched-chain essential amino acid (BCAA) leucine [52-54] (Figure 2). Recent advances in molecular biology have elucidated two parallel mechanisms of mTORC1 activation: 1) the upstream activation of the small GTPase *Rheb* (Ras homolog enriched in brain) by growth factor signals (insulin, IGF-1, PDGF) and high cellular energy levels (glucose, ATP), and 2) the amino acid-dependent translocation of *inactive mTORC1* to active Rheb localized at late endosome or lysosome compartments [53-56] (Figure 2). The activity of Rheb is tightly regulated by the tuberous sclerosis proteins TSC1 (hamartin) and TSC2 (tuberin), which form a functional heterodimeric complex. TSC1 stabilizes TSC2 that possesses a GTPase-activating protein, which hydrolyses GTP to GDP. The TSC1/TSC2 complex provides this function to Rheb leading to inactivation of Rheb. In contrast, insulin and IGF-1, both activate the kinase Akt (protein kinase B) as well as other growth-related kinases such as ERK and RSK, which phosphorylate TSC2 and thereby attenuate the inhibitory function of the TSC1/TSC2 complex. This inhibition leads to activation of Rheb with final activation of mTORC1 [57-60] (Figure 2). 

Besides the important input of growth factor signaling on mTORC1 activation, AMPK, an essential energy sensor, plays a key role in energy-dependent mTORC1 regulation. During states of energy-deficient conditions like glucose deprivation, ATP levels fall and AMP levels rise, resulting in AMPK activation. AMPK phosphorylates TSC2 and Raptor, thereby suppressing mTORC1 activity [60,61]. Abundant cellular energy provided by hypercaloric and high glycemic loads of Western diet thus reduces AMPK activity and stimulates mTORC1 signaling. In Western diet hyperglycemic food compounds are frequently combined with dairy products like cornflakes with milk or pizza and burgers combined with cheese.

Notably, leucine has been identified as the primarily responsible branched-chain amino acid for mTORC1-dependent stimulation of skeletal muscle protein synthesis [52]. In fact, in response to amino acid depletion, mTORC1 activity is rapidly abolished [62]. Amino acid starvation impairs binding of mTORC1 to Rheb [63]. From all essential amino acids, leucine exerts the greatest effects on mTORC1 signaling [42,48,51,62]. Recent evidence has been provided that amino acids and especially leucine promote the cellular translocation of inactive mTORC1 to lysosomal compartments enriched in activated Rheb [53,55]. This spatial regulation of inactive mTORC1 by amino acids is mediated by an active *Rag heterodimer*, which is of utmost biological importance for amino acid sensing by mTORC1. Thus, mTORC1 integrates not only growth factor and energy derived signals towards Rheb, but requires a parallel signal input of leucine for final mTORC1 activation by translocation of inactive mTORC1 to cell compartments enriched in activated Rheb (Figure 2). These two independent major pathways of mTORC1 activation explain why either insulin and IGF-1 signaling or amino acid signaling alone is not sufficient to reach maximal mTORC1 activation. Insulin is not able to activate the mTORC1 pathway when cells are deprived of amino acids [64]. Indeed, both insulin- and amino acid signaling are required for maximal mTORC1 activity [65]. Evidence will be provided that only milk proteins in comparison to meat and fish have the unique ability to preferentially increase both the insulin/IGF-1 and the leucine signaling pathways necessary for maximal mTORC1 activation.

Growing cells and especially proliferating tumor cells not only require increased amounts of amino acids and protein but also high amounts of lipids to enlarge their cellular membrane compartments. It is thus not surprising that the key transcription factor of lipid biosynthesis SREBP-1 (sterol regulatory element binding protein-1) is dependent on upstream activation of mTORC1 [66,67].

### Oncogenic mTORC1-signaling in prostate cancer cells

**Figure 3:**
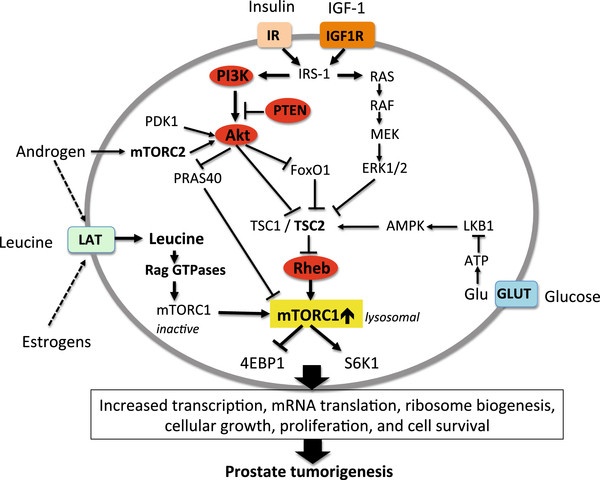
** Aberrations of PI3K-Akt-mTORC1 signaling pathway in prostate cancer.** Mutation-induced hyperactivated PI3K, Akt and overexpression of Rheb stimulate mTORC1. Loss of PTEN results in hyperactive Akt signaling. Androgens activate Akt via mTORC2 and via upregulation of L-type amino acid transporters (LAT) promote leucine-stimulated mTORC1 activation.

Increased insulin, IGF-1 and especially leucine signaling with activation of mTORC1 are not only a requirement for physiological growth during the neonatal period and puberty but play a pivotal role in the process of tumorigenesis [43,68-74]. In addition to various other cancers, signaling pathways that activate mTORC1 are frequently deregulated in PCa [43,74,75]. mTORC1 is upregulated nearly in 100% of advanced human PCa [76] (Figure 3). Activation of mTORC1 is a central component downstream of the phosphatidyl-inositol-3-kinase (PI3K)/Akt signaling cascade. Akt-mediated phosphorylation of TSC2 prevents TSC1/TSC2 complex formation, which drives the small GTPase Rheb into the GTP-bound active state [77], leading to the phosphorylation and activation of mTORC1 at Ser2448 [78,79]. PTEN, phosphatase and tensin homologue deleted on chromosome 10, is a negative regulator of Akt activation, as it converts phosphatidylinositol 3,4,5-triphosphate [PtdIns(3,4,5)P_3_ back to PtdIns(4,5)P_2_, leading to reduced recruitment of Akt to the cell membrane, which is the important location for PDK-1-mediated final phosphorylation and full activation of Akt [80]. The PI3K/Akt/mTORC1-pathway is aberrantly activated in the majority of PCa due to PTEN copy number loss or loss of function of at least one PTEN allele [75,76,81]. Genetic findings in mouse models implicate mTORC1 hyperactivation in both PCa initiation and progression [82-87]. It has recently been reported that Rheb, the final upstream activator of mTORC1, is amplified in human PCa [88]. In the mouse prostate, *Pten* haploinsufficiency cooperates with Rheb-overexpression and markedly promoted mTORC1-mediated prostate tumorigenesis [88] (Figure 3). Compelling evidence has recently demonstrated that mTORC1 controls the translational program of mRNA subsets of genes involved in PCa initiation, invasion and metastasis [89-91]. 

### Cross-talk between androgen receptor and mTOR in prostate cancer

The two most frequently activated signaling pathways in PCa are driven by androgen receptor (AR) and PI3K. Genetic loss of either *mTOR* or *Akt1* is sufficient to signifcantly reduce initiation of PCa in the conditional Pten mouse model [90-92]. The allosteric mTORC1 inhibitor rapamycin has been shown to revert Akt-dependent early prostate intraepithelial neoplasia (PIN) in young mice through regulation of apoptotic and HIF-1-dependent pathways [93]. It has been shown in the prostate-specific *Pten*-deleted mouse model of PCa that depletion of androgens significantly inhibited tumor growth progression without altering the activation of Akt and mTOR, however, the combination of antiandrogen treatment with rapamycin-mediated mTORC1 inhibition exhibited additive antitumor effects [94]. Recent evidence points to a reciprocal feedback regulation of PI3K and AR singaling in *PTEN*-deficient PCa [95]. In human and murine tumors with *PTEN* deletion inhibition of the PI3K pathway promoted AR activity, whereas androgen blockade activated Akt signaling [95]. Combined PI3K and AR inhibition was superior to single agent therapy in PTEN loss PCa [95].

Dihydrotestosterone (DHT)-stimulation of LNCaP prostate cancer cells, which have constitutive PI3K/Akt pathway activation due to loss of PTEN, caused increased expression of cyclin D_1_, D_2_, and D_3_ proteins, retinoblastoma protein hyperphosphorylation and cell cycle progression [96]. DHT treatment increased mTORC1 activity as assessed by phosphorylation of the downstream targets S6K1 and 4EBP1. However, mTORC1 inhibition with rapamycin blocked the DHT-stimulated increase in cyclin D proteins [96]. mTORC1 activation by DHT was dependent on AR-stimulated mRNA synthesis, including genes that regulate cellular leucine availability [96]. L-type amino acid transporters such as LAT1 and LAT3 mediate the uptake of essential amino acids, especially leucine, for leucine-mediated mTORC1 signaling promoting cell growth and proliferation of PCa cells [97]. In the androgen-sensitive LNCaP cell line, androgen-induced upregulation of LAT3 expression was related to increased leucine-mTORC1-mediated tumor cell growth [97]. High levels of LAT3 were detected in primary disease, whereas increased levels of LAT1 were observed after ADT and in metastatic lesions [97]. It has recently been shown that BCAA metabolism is affected by malignant progression of PCa cells [98]. Androgens rapidly reduced the protein concentration of the cell cycle inhibitor p27 in PCa cells by increasing proteasome-mediated degradation of p27 [99]. Androgens increased mTORC2-mediated Akt(S473) phosphorylation, which stimulated Akt-mediated phosphorylation of p27(T157), the critical signal for p27 proteasomal degradation [99]. Furthermore, androgen-mTORC2-mediated activation of Akt resulted in increased phosphorylation of FoxO1, which in its phosphorylated form is extruded from the nucleus into the cytoplasm [99]. Activated Akt is part of the canonical pathway activating mTORC1. Furthermore, cytoplasmic FoxO1 has been shown to bind to the C-terminal sequence of TSC2, thereby disrupting the TSC1/TSC2 complex resulting in further activation of mTORC1 [100]. Moreover, androgen-mediated nuclear FoxO1 extrusion resulted in diminished expression of the AMPK activator Sestrin3, thus allowing further activation of mTORC1 [101].

Recently, Hsieh *et al.*[91] have demonstrated that complete mTOR inhibition by the ATP site inhibitor of mTOR, INK128, prevented PCa invasion and metastasis *in vitro*. Obviously, mutations or activating posttranslational modifications of the upstream activators of mTORC1 (Akt, PI3K, Rheb, and loss of PTEN function) amplify oncogenic mTORC1 signaling in PCa cells (Figure 3), which drives the ´cancerous´ translation machinery steering cancer initiation, cancer invasion and metastasis [91]. Thus, substantial evidence underlines the pivotal role of exaggerated mTORC1 signaling in both PCa development and progression. Continuously increased mTORC1 signaling has been associated with tumor initiation as well as tumor progression [91]. In fact, sustained proliferative signaling has been identified as a hallmark of cancer and comprises the most important biological capability during the multistep development of human tumors [102].

### Mammalian milk: an endocrine mTORC1 activating system for neonatal growth

To understand the impact of dairy protein consumption on PCa tumorigenesis, we have to appreciate the signal transduction of mammalian milk. Although the growth-stimulating effect of cow´s milk has been recognized since 1928, from it´s effect on childhood stature, the growth-promoting pathways of milk-derived signal transduction have not been a matter of medical concern [103]. There is substantial epidemiological evidence that cow´s milk consumption increases linear growth and body mass index in infants and accelerates the onset of menarche [104-106]. The function of mammalian milk is not only to provide sufficient calories and nutrients to the newborn but in parallel to promote postnatal growth by milk-mediated growth factor signaling. This overlooked growth-promoting functionality of milk represents the fundamental obstacle, which for many decades obscured the link between the growth-stimulating activity of cow´s milk and cow´s milk-mediated promotion of cancer growth. To fulfill its biological function to promote growth and survival, milk enhances the activity of mTORC1 by increasing 1) insulin/IGF-1 signaling leading to activation of Rheb, and 2) by providing substantial amounts of the essential BCAAs like leucine, which stimulate amino acid-mediated mTORC1 activation.

### Differences in the magnitude of mTORC1 signaling of cow´s milk versus human milk

**Figure 4:**
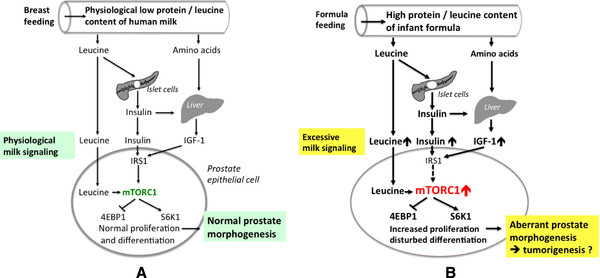
** Milk signaling, prostate gland morphogenesis and tumorigenesis.A**) Signaling of human milk and physiological prostate morphogenesis. **B**) Excessive cow´s milk signaling by infant formula feeding disturbing regular prostate morphogenesis resulting in long-term adverse effects promoting prostate tumorigenesis.

For adequate species-specific growth requirements, each mammalian species has developed its own species-specific magnitude of milk-mediated mTORC1 signaling. The strength of mTORC1-mediated stimulation of mammalian growth is associated with the total protein and total leucine concentration in mammalian milk [107]. The milk of various mammalian species shows significant species-dependent variations in the concentration of total milk protein. However, milk of all mammals exhibit a constant ratio of 0,1 g of leucine per 1 g of total milk protein [108]. Remarkably, species with the highest milk protein concentration exhibit the most rapid growth rate. An inverse relation exists between the protein plus leucine content of mammalian milk and the neonate´s rate of birth weight duplication [107]. For instance, the leucine content of rat´s milk is 11 g/L, of cat´s milk 8,9 g/L, of cow´s milk 3,3 g/L , and of human milk 0,9 g/L, respectively [108]. The rat doubles birth weight already after 4 days, the cat after 10 days, the calf after 40 days, and the human neonate, the mammal with the slowest growth rate, after 180 days, respectively [107]. These data imply, that the leucine-mTORC1 signaling axis of cow´s milk is much higher than that of human milk, as cow´s milk contains threefold the amount of total protein and total leucine in comparison to human milk [107,108]. Moreover, the weight gain of calves during the first year of cow´s milk feeding (0.7-0.8 kg/day) is nearly 40-times higher than that of breast-fed human infants (0.02 kg/day) [109]. It has been demonstrated that cow´s milk-based infant formula feeding significantly increases serum concentrations of leucine, insulin, and IGF-1 in comparison to physiological breast-feeding [110,111] (Figure 4). Thus, cow´s milk-based infant formula feeding induces higher magnitudes of mTORC1 signaling in comparison to natural breast-feeding of human milk [112,113]. The great differences in the magnitude of mTORC1 signaling may explain the differences in growth velocity and weight gain between *Bos taurus* and *Homo sapiens*. Moreover, the relatively low mTORC1 signaling axis of human neonates in comparison to *Bos taurus* allows a slower rate of brain growth permitting more time for the accumulation of long-chain omega-6 polyunsaturated fatty acids (specifically 20:4n6) and long-chain omega-3 polyunsaturated fatty acids (specifically 22:6n3), critical nutrients for the development of complex brain functions, an evolutionary advantage of *Homo sapiens*[114]. With regard to mTORC1-dependent postnatal events in prostate morphogenesis and prostate gland development it is of special concern that infant formula feeding in comparison to natural breast-feeding may over-stimulate mTORC1 signaling thereby disturbing physiological conditions of prostate growth and development during the postnatal period.

### Cow´s milk protein elevates IGF-1 serum levels: a known risk factor of prostate cancer

Insulin-like growth factor-1 (IGF-1) is an important activator of PI3K/Akt signaling resulting in mTORC1 activation [57,60,68]. It has been demonstrated in rat hepatocyte primary cultures that hepatic IGF-1 gene expression directly depends upon the availability of essential amino acids [115]. Elevated IGF-1 serum concentrations are associated with consumption of animal versus vegetable proteins. In comparison to meat protein increased intake of milk protein by milk consumption from 200 to 600 ml resulted in a 30% increase in IGF-1 serum concentrations [116]. Furthermore, skim milk protein versus meat intake increased serum IGF-1 concentrations in 8-year-old boys [117], underlining the higher IGF-1-enhancing activity of milk proteins in comparison to meat protein. In prepubertal boys, casein versus whey protein elicited a greater rise in plasma IGF-1 concentrations, whereas whey protein versus casein elicited a greater rise in postprandial insulin plasma concentrations [118]. Thus, there is substantial evidence in humans, that cow´s milk protein intake in comparison to meat protein exerts stronger effects on protein-induced IGF-1 signaling. Therefore, it is reasonable to differentiate between *signaling proteins* like milk proteins and *structural proteins* like meat and fish protein, which are less efficient in elevating insulin and IGF-1 plasma concentrations than milk proteins.

Lower IGF-1 signaling due to mutations with reduced activity of the IGF-1 receptor are associated with longevity and lower incidence rates of cancer [119]. Moreover, congenital IGF-1 deficiency has been shown to confer protection against the development of malignancies in humans including a lower prevalence of PCa [120]. In a cohort of 99 untreated individuals with Laron syndrome, who exhibit growth hormone receptor mutations with congenital IGF-1 deficiency, only one nonlethal malignancy has been detected after 22 years of monitoring, whereas not affected relatives exhibited a cancer prevalence of 17% with PCa being the third most common cancer in this control population [121]. Cells cultured with IGF-1-deficient serum of Laron individuals showed a 20% reduction of TOR expression and exhibited increased activity of nuclear FoxO protein levels [122]. Thus, low IGF-1 serum levels attenuated IGF-1/mTORC1-mediated cell proliferation and increased FoxO-mediated anti-oxidative responses by upregulation of superoxide dismutase and catalase as well as by FoxO-driven pro-apoptotic responses, all of which are most important cancer-preventive mechanisms [122]. In accordance with observed cancer-protective effects of low IGF-1 signaling is a recent epidemiological study, which demonstrated that high serum IGF-1 concentrations were associated with increased cancer deaths in older men [123]. Notably, men with type 2 diabetes were found to have a lower incidence of PCa, presumably due to decreased IGF-1 serum levels [124]. However, recent large cohort studies have reported the association of type 2 diabetes with advanced high-grade PCa [125]. Data from meta-regression analyses of case control studies have provided substantial evidence that high serum concentrations of insulin and IGF-1 are associated with increased risk of PCa [126-128]. Increased serum concentrations of IGF-1 are specifically involved in the early pathogenesis of PCa [126]. *The European Prospective Investigation into Cancer and Nutrition* clearly confirmed the correlation between increased dairy protein consumption and raised IGF-1 serum concentrations in adults [129,130]. A recent cross-sectional analysis using data of 1,798 men in the UK demonstrated that for one standard deviation increase in dairy protein intake, IGF-1 increased by 6.02 ng/ml [131]. Among other animal- and plant-derived proteins, dairy protein was most strongly associated with increased IGF-1 serum concentrations [131]. Notably, low versus high meat intake was not associated with a change of IGF-1 serum levels [131]. These data strongly imply, that the increase of serum IGF-1 concentrations by cow´s milk protein consumption may result in elevated IGF-1-driven mTORC1 signaling, the driving mechanism promoting the initiation and progression of PCa.

### Cow´s milk protein intake increases postprandial serum insulin levels

Insulin, like IGF-1, is a growth hormone, which activates insulin receptor as well as IGF-1 receptor signaling and stimulates the activity of mTORC1 [68,132]. As it is the biological function of mammalian milk to promote growth, milk performs its mission by increasing the serum concentrations of both growth hormones insulin and IGF-1. Cow´s milk proteins, which are absent in Paleolithic diets and traditional diets of Southeast Asia, significantly contribute to higher insulin/IGF-1 signaling of Western diet [133]. Mammalian milk upregulates insulin secretion of pancreatic β-cells as well as IGF-1 synthesis by the liver (Figure 4). BCAAs, predominantly leucine, stimulate β-cell mTORC1 resulting in increased insulin secretion [134-137]. Moreover, milk consumption not only activates the somatotropic axis but also the entero-insular axis by stimulating intestinal incretin secretion of glucose-dependent insulinotropic polypeptide (GIP), which further augments insulin secretion [138,139]. Milk´s excessive insulinotropic activity is well characterized by cow milk´s high *insulinemic index*[140].

It has recently been confirmed that BCAA-enriched postprandial serum after ingestion of whey protein by healthy individuals significantly induced insulin secretion of isolated mouse pancreatic islets [141]. The insulinogenic effect of whey protein was predominantly mediated by the whey protein-derived amino acids leucine, isoleucine, valine, lysine and threonine and was augmented by increased intestinal release of GIP [141]. Casein protein hydrolysate and leucine co-ingestion in patients with type 2 diabetes stimulated postprandial insulin secretion [142]. Notably, increased daily intake of milk but not meat significantly elevated fasting insulin and IGF-1 serum concentrations and increased insulin resistance in 8-year old boys [117,143]. Milk-induced insulin resistance can be well explained by increased mTORC1-mediated activation of S6K1. Insulin receptor substrate-1 (IRS-1) is an important target of S6K1 phosphorylation, which inhibits downstream insulin signaling in adipose tissue, liver and skeletal muscle [144] (Figure 2).

In accordance with epidemiological evidence, an association of diet-induced hyper-insulinemia with accelerated growth of LNCaP xenografts has been reported [145]. Moreover, the insulin-analogues glargine and detemir exhibited IGF-1-like mitogenic and anti-apoptotic activities in cultured PC-3 prostate cancer cells [146]. In contrast, reduced circulating IGF-1 levels obtained by calorie restriction attenuated mTORC1 signaling and inhibited tumorigenesis [147]. Dietary restriction in mice resulted in reduced IGF-1 serum concentrations and decreased mTORC1 activity in the dorsolateral prostate of mice [148]. Recent evidence supports a “higher-level” interaction between AR signaling and IGF-1 signaling via recruitment of direct pathways toward activation, transcriptional regulation, and protein posttranslational changes, all critical to PCa cell survival [149]. The reduction in high protein intake commonly provided by Western diet has been proposed as an important strategy for dietary anti-cancer interventions [150]. However, the quantity and quality of protein intake has been recognized as the key determinant of circulating IGF-1 concentrations and postprandial hyperinsulinemia. Milk (insulinemic index > 100) and especially the whey proteins are highly insulinotropic in comparison to meat (insulinemic index 51) [140]. Moreover, total protein intake is not the most important determinant controlling high IGF-1 serum concentrations as milk protein induces higher IGF-1 concentrations than meat. Thus, for understanding the role of nutrient signaling in cancer biology it appears to be of pivotal importance not only to consider total daily protein intake but rather to differentiate between the intake of BCAA-enriched *signaling proteins* (derived primarily from milk) and the intake of less insulinotropic *structural proteins* (primarily derived from meat and fish).

### Whey protein ingestion activates mTORC1 signaling

There is yet no direct experimental evidence for the activation of mTORC1 in prostate epithelial cells after cow´s milk protein consumption. However, it has been convincingly demonstrated that a single dose (26.6 g) of whey protein isolate in young men immediately after a resistance exercise significantly increased mTORC1 signaling in skeletal muscle (*vastus lateralis*) 2 h post-exercise [151]. Whey protein intake significantly enhanced the phosphorylation of mTOR(Ser2448), 4EBP1(Thr37/46) and S6K1(Thr389) at 2 h post-exercise [151]. These data are in agreement with studies in rat skeletal muscle, which have demonstrated a dose-response between leucine ingestion and increased S6K1(Thr389) phosphorylation [152]. In addition to S6K1 phosphorylation, the phosphorylation of 4EBP1 is also sensitive to amino acid provision [153], and the site-specific phosphorylation of Thr37/46 is reported to be crucial for optimal activation of 4EBP1 [154]. These data of whey protein-induced mTORC1 activation in muscle cells are most likely conferrable to other human tissues like the prostate gland and support the role of dairy protein-induced mTORC1 signaling as a fundamental biological function of mammalian milk to promote growth.

### Milk-mediated mTORC1 signaling amplifies oncogenic mTORC1 signaling

Most importantly, to achieve the physiological requirements for adequate growth, milk proteins provide the highest amounts of leucine and the most effective BCAAs required for mTORC1 activation [48,51,52,62]. Whey proteins have to be regarded as unique life starter proteins that contain the highest amount of leucine (14%), followed by casein (10%), the major protein constituent of cow´s milk and cheese [155]. Since mTORC1 signaling positively regulates protein synthesis and ribosome biogenesis, both of which require amino acids, it makes physiologically sense that mTORC1 signaling is highly dependent upon amino acid availability. Withdrawal of leucine has been shown to be nearly as effective in down-regulation of mTORC1 signaling as withdrawal of all amino acids [52,62]. Moreover, the preeminent effect of leucine withdrawal has been consistently observed in a variety of cell types, thus underlining the primacy of leucine in amino acid-mediated mTORC1 regulation [47-49].

It is important to realize that milk protein intake appears to be more efficient in activating mTORC1 than meat or cheese protein consumption as milk contains the highly water soluble and easily hydrolysable leucine-rich whey proteins, which are responsible for the rapid and elevated postprandial rise in plasma leucine concentrations [139,141]. These whey protein-mediated high pulsatile elevations of plasma leucine- and GIP concentrations may explain the high insulinemic index of milk and other whey protein-containing milk products like yoghurt (insulinemic index of 89-115) in comparison to energy-equivalent protein uptakes of beef (insulinemic index of 51) and cheese (insulinemic index of 45) [156]. In comparison to meat, intake of milk and whey protein-comprising milk products thus results in higher and more rapid increases of leucine and insulin signals, which are both important downstream activators of mTORC1. As signal transduction in biological systems is known to respond more efficiently to sudden increases of incoming signals (Δ signal) than to prolonged exposures of high signal levels, the pronounced differences in the leucine-mTORC1-insulin kinetics between whey protein and meat may explain why Paleolithic type diets are less insulinotropic and presumably less effective in mTORC1 stimulation than whey protein-accentuated Western diets [157]. Previously, the growth-promoting effects of milk consumption was explained exclusively by milk´s ability to induce higher plasma concentrations of IGF-1 while ignoring the co-stimulatory effects of leucine, insulin, and IGF-1 signaling, all of which are finally integrated by the central regulator mTORC1 [158]. In this regard, meat and fish protein consumption, in comparison to whey protein-enriched dairy products, may exert less oncogenic stimulation. Circulating pulsatile rises of leucine and insulin concentrations together with permanently elevated serum concentrations of IGF-1 may be responsible for cow milk´s optimized mode of hyperactivated mTORC1 signaling, which promotes growth stimulation of prostate epithelial cells and further amplifies already upregulated oncogenic mTORC1 signaling of PCa cells with preexisting alterations of cell growth-promoting signaling pathways (Table 1; Figure 4B). 

**Table 1:** **Comparison of leucine-insulin-IGF-1-signaling between dairy-derived signaling proteins versus meat-derived structural proteins**[116-118]

**Protein source**	**Leucine**	**Insulin**	**IGF-1**
Whey	↑↑↑	↑↑↑	
Casein	↑↑		↑↑↑
Milk protein	↑↑	↑	↑↑↑
Meat protein	↑↑	↑	↑

### BCAA and leucine overload by dairy protein-rich Western diet

A worldwide continuous increase of dairy protein consumption has occurred in Western countries, as exemplified by the annual increase of cheese consumption per capita in the Federal Republic of Germany from 3.9 kg in 1935 to 23.0 kg in 2011 (Figure 1) [112]. The high BCAA and leucine content of animal protein-rich Western diet strongly contrasts to the low dietary leucine amounts in traditional Asian diets because of their high reliance upon plant proteins, containing little leucine. Thus, during the last five decades a steady increase in total leucine intake in westernized countries has occurred as exemplified by the German per capita leucine consumption (Table 2). The abundant cheese consumption in Western countries substantially contributed to the increased total leucine intake, which has been recognized as an important activating signal of mTORC1 and may explain the association between cheese consumption and increased risk of PCa [6]. For example, the leucine content of 100 g of Gouda cheese (2.4 g leucine) is considerably higher than that of 100 g white cabbage (0.056 g), or 100 g apple (0.016 g). To reach the leucine content provided by 100 g Gouda cheese, 4.2 kg of white cabbage or 100 apples would have to be consumed [112]. Cheese is widely consumed with fast food products like pizza, tacos and cheeseburgers. The abundance of leucine in the Western diet and especially the combination of easily releasable leucine from whey proteins in combination with high glycemic load foods may thus promote mTORC1-driven prostate tumorigenesis. The introduction of widespread refrigeration technology since the 1950s in industrialized countries has allowed for the increased and persistent availability of dairy proteins. 

**Table 2:** Annual animal protein-derived per capita leucine intake in Germany

**Animal protein [g/y]**	**1950/51**	**1974/75**	**2007/08**
Total meat (≈21 g Leucine/kg)	552	1 172	1 268
Egg/egg products (≈13 g Leucine/kg)	98	225	169
Fish (≈19 g Leucine/kg)	131	78	143
Cheese (≈25 g Leucine/kg)	96	293	558
Average total per capita leucine uptake [g/y]	877 (100%)	1768 (199%)	2138 (241%)

### Commercial cow´s milk: a source of dietary estrogens

**Figure 5:**
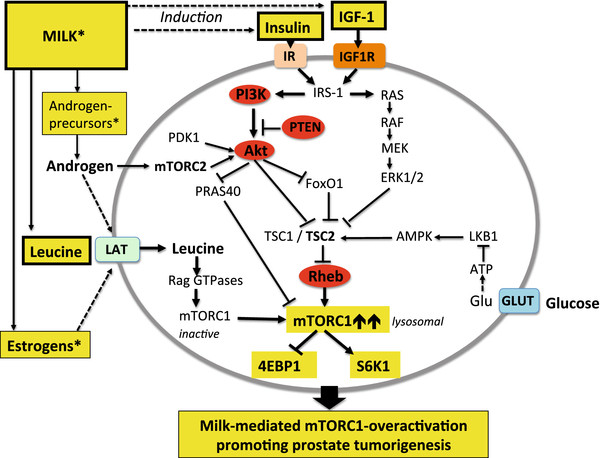
** Boostering effects of cow´s milk consumption on prostate cancer-associated high mTORC1 signaling.** Milk and dairy protein consumption increase serum insulin and IGF-1 concentrations and provide abundant leucine for mTORC1 activation. Milk-derived estrogens and androgen-precursors from pregnant* cows augment LAT-mediated leucine uptake promoting further mTORC1 activation. Milk signaling shares common pathways of PCa with hyperactivated mTORC1 signaling due to cancer-associated alterations of Akt, PI3K, PTEN and Rheb (labeled in red).

There is recent concern that not only androgens, but also estrogens promote PCa [159]. Commercialized milk production by pregnant cows releases uncontrolled amounts of bovine steroids into the human food chain. PCa cells express estrogen receptors (ERs). ADT has been shown to upregulate stromal ERs [160]. The addition of estradiol (10^-8^ M) significantly promoted growth of LNCaP cells *in vitro*[41]. In industrialized countries, a significant percentage of commercial milk comes from pregnant cows containing elevated concentrations of estrogens and progesterone [161-164], which may synergistically enhance mTORC1 signaling. 5α-Pregnanedione, a steroid compound of commercial cow´s milk, is a direct precursor of DHT and may act through mTORC2-Akt as well as AR-LAT3-leucine-mediated activation mTORC1 in prostate tumorigenesis (Figure 5). Even more evidence underlines the role of estrogens in prostate tumorigenesis [165,166]. Animal and limited human studies suggest that estrogens are involved in prostate carcinogenesis by genotoxic mechanisms [166]. 17β-estradiol, which is generated from testosterone by the enzyme aromatase, can be converted to catecholestrogens, which through redox cycling may generate reactive metabolites that can adduct to DNA and potentially lead to mutations [166]. Furthermore, developmental studies of prostate morphogenesis demonstrated a critical time for estrogen action during the development of the prostatic tissue [165]. It has been suggested that estrogen-sensitive cells may remain in the prostate and be more responsive to estrogens later in life or be less responsive to the normal controlling mechanisms of prostatic growth [165]. Dogs as with men are able to develop PCa. Marked prostate hyperplasia has been induced in dogs especially by combined administration of DHT with estrogens [167,168]. 

In the prostate, ER-β is preferentially expressed in epithelial cells but ER-α is found in stromal and basal cells [169]. There is evidence for increased ER staining intensity in malignant prostates [170]. Recently, ER-β expression has been detected in PCa cells [171]. Notably, in the human breast cancer MCF-7 cell line, 17β-estradiol stimulated leucine uptake by increasing the expression of LAT1 [172]. Although no data are yet available on estrogen-mediated leucine uptake in PCa cells, it is conceivable that commercial milk-derived estrogens may further augment leucine-mTORC1-signaling of PCa cells. Remarkably, cow´s milk feeding to female rats with chemically dimethylbenz (a) anthracene (DMBA)-induced mammary cancer in comparison to milk-free controls promoted the growth of mammary tumors and doubled the number and volume of tumor cells [173,174]. Moreover, commercial cow´s milk was found to be uterotrophic in rats [175], which represents more evidence of milk´s estrogenic and pro-proliferative activity. Remarkably, 17β-estradiol stimulated leucine uptake by MCF-7 breast cancer cells, which appear to increase leucine uptake predominately by LAT1 [172]. PCa cells coordinate the expression of L-type amino acid transporters such as LAT1 and LAT3 to maintain sufficient levels of leucine needed for mTORC1 signaling and cell growth [97]. Noteworthy, the mTORC1 inhibitor rapamycin inhibited the proliferation of ER-positive breast cancer cells [176]. These animal studies and one human intervention study [164] strongly suggest that commercial cow´s milk (especially if it is derived from pregnant cows) contains estrogen metabolites with significant oral bioavailability. This observation is further supported by evidence showing that the most prevalent form of estrogen in cow´s milk is estrone sulfate [159], which coincidentally comprises almost half of the conjugated estrogens in the most frequently prescribed oral hormone replacement therapies for menopausal women.

It should be realized that low fat (skim milk) was as effective in mammary tumor promotion as whole milk [173,174]. Although it is well known that the lipid soluble steroids of milk are mainly distributed within milk´s fat fraction, conjugated estrogens have been found in high amounts in skim milk [162]. In fact, several studies emphasized a stronger association of PCa with the consumption of low fat/nonfat milk than with whole milk [16,18,19]. Hence, it is conceivable that the protein fraction of milk may act synergistically with conjugated estrogens present in commercial skim milk in the promotion of prostate tumorigenesis.

Moreover, Ganmaa *et al.*[6] provided evidence that the combination of “milk + cheese” was associated with increased risk of PCa mortality, a combination that is subsumed as “total dairy” in various studies [10]. Cheese consumption is a major dietary source of milk fat, which is contaminated by pregnancy-derived bovine estrogen metabolites in free and conjugated forms [161,162]. Excessive cheese consumption derived from commercial milk of pregnant cows thus provides an undesirable estrogen load, which may synergistically amplify the intrinsic growth promoting effects of milk proteins. Hence, substantial evidence supports the view that milk signaling shares synergistic effects with oncogenic mTORC1 signaling pathways of PCa. Commercial milk intake by increasing plasma concentrations of insulin, IGF-1, leucine, estrogens and androgen-precursors may amplify preexistent high mTORC1 signaling due to genetic alterations of the PI3K/PTEN/Akt/Rheb/mTORC1 pathway of PCa cells (Figure 5).

### Obesity, insulin resistance and increased mTORC-1 signaling and prostate cancer

Increased cow´s milk signaling via insulin, IGF-1 and leucine has recently been linked to dysregulated early metabolic programming and early promotion of mTORC1-mediated adipogenesis [112,113]. More evidence supports the influence of diet and obesity on prostate health [177]. However, only an overall weak association of PCa risk with obesity has been reported [178,179]. Nevertheless, obesity appears to be associated with more advanced, higher-grade disease and PCa mortality [179-181]. Indeed, a relationship between BMI and advanced PCa has been established [181,182]. Higher milk consumption in children has been associated with increased BMI [105]. It has been recognized that leucine mediates mTORC1 signaling of adipocytes and plays a crucial role in adipogenesis, adipogenic action of PPARγ and SREBP-1-mediated lipid synthesis [112,113,183-189]. Moreover, leucine-stimulated mTORC1-S6K1 signaling has been shown to play an important role in the induction of insulin resistance [144,190] (Figure 2). In humans, insulin resistance was induced by infusion of high concentrations of amino acids, whereas the mTORC1 inhibitor rapamycin improved insulin action [191]. Infusion of an amino acid mixture to healthy men resulted in elevation of plasma amino acids, hyperinsulinemia and marked activation of S6K1 with increased inhibitory IRS-1 phosphorylation [192] (Figure 2). In comparison to normal subjects, oral doses of leucine (0,2 g/kg) stimulated exaggerated insulin release and induced basal hyperinsulinemia in obese patients [193].

Notably, milk consumption in prepubertal boys has been shown to increase serum insulin concentrations associated with the induction of insulin resistance [143]. The resulting hyperleucinemia and hyperinsulinemia may exert leucine- and insulin-mediated stimulatory effects of prostate mTORC1 signaling. In fact, insulin as well as hyperinsulinemic rat serum after generation of high fat diet-induced insulin resistance promoted growth of androgen-independent prostate cancer PC-3 cells [194]. Furthermore, obesity-related higher concentrations of circulating estrogens in synergy with commercial milk-derived estrogens may stimulate leucine uptake of prostate cells thus orchestrating a pro-proliferative metabolic environment enhancing mTORC1 signaling. Moreover, obesity-induced insulin resistance is not only a proliferative risk constellation mediated by hyperinsulinemia but is also associated with elevations in BCAAs including leucine [195,196]. In obesity, adipose tissue down-regulates BCAA uptake and metabolism resulting in elevated concentrations of circulating BCAAs [197,198]. In obesity and states of insulin resistance, elevated BCAA have been associated with reduced expression of two adipose tissue BCAA catalytic enzymes: mitochondrial branched-chain aminotransferase 2 (BCAT2) and branched-chain α-keto acid dehydrogenase (BCKD E1α subunit) complex [197-199]. Thus, high milk and dairy protein consumption in obese men may have additive effects on already elevated obesity-derived leucine levels, which may further enhance mTORC1 activity of prostate epithelial cells and PCa cells.

### Androgen deprivation therapy down-regulates LAT3-mediated leucine uptake

PCa cells coordinate the expression of cellular amino acid transporters LAT1 and LAT3 to maintain sufficient levels of leucine required for mTORC1 signaling and cell growth [97]. ADT downregulates AR-mediated expression of LAT3, which is important to maintain intracellular leucine levels for mTORC1 activation. Low levels of intracellular leucine activate the translation of the transcription factor ATF4, which initiates the transcription of LAT1 to restore leucine-mediated mTORC1 signaling [97]. Inhibition of either LAT3 or LAT1 can lead to decreased growth of PCa cells [97]. Thus, it is conceivable, that the total dietary leucine influx as wells as androgen- and estrogen-regulated intracellular leucine-uptake mechanisms play important roles for the regulation of leucine-mediated mTORC1 signaling. These findings suggest that preliminary therapeutic responses to ADT may be related to attenuation of androgen-dependent leucine-mediated activation of mTORC1 [97], a therapeutic mechanism, which may be counterbalanced by high intake of commercial cow´s milk protein.

### Leucine and mTORC1-dependent inhibition of autophagy

Autophagy is an evolutionarily conserved catabolic pathway that has multiple roles in carcinogenesis and cancer therapy [200]. Autophagy can inhibit the initiation of tumorigenesis through limiting cytoplasmic damage, genomic instability and inflammation. Loss of certain autophagic genes can lead to cancer [200]. Autophagy is connected to major cancer networks and is predominately regulated by the activity of the mTORC1 pathway [200]. Pathway enrichment analyses recently revealed that mTORC1 and insulin signaling pathways are important in the regulation of genes involved in autophagy [201]. Aberrant autophagy has been implicated to play a major role in tumorigenesis [202,203]. Activated mTORC1 stimulates cell growth and cell proliferation, whereas suppression of mTORC1 activates autophagy [204]. Autophagy is an important catabolic process involving lysosomal turnover of proteins and organelles for maintenance of cellular homeostasis and mitigation of metabolic stress. Autophagy defects are linked to aging and cancer. Autophagy is often impaired in human PCa due to either activation of the PI3K/Akt/mTORC1 pathway, which inhibits autophagy, or through allelic loss of the essential autophagy gene *beclin 1*[205-207]. ADT induces apoptosis, as well as autophagy in androgen-responsive PCa cells [208]. Amino acids and leucine in particular are involved in the regulation of autophagosome formation [209]. Emerging evidence has linked leucine deprivation-induced protein breakdown to autophagy. It has recently been shown, that leucine inhibits autophagy in an mTORC1-dependent manner [210]. It is conceivable that a high dietary influx of leucine may adversely affect autophagy regulation in prostate epithelial cells. Impaired autophagy regulation of prostate epithelial cells and PCa cells by high dairy protein consumption may thus further promote tumorigenesis and may augment aggressive transformation of PCa cells, especially in those PCa cells exhibiting an allelic loss of beclin-1. It has been shown that mTORC1 inhibition by rapamycin induced autophagy and radiosensitized PTEN null PCa cells [211]. Furthermore, the red yeast rice-derived compound monascuspiloin in combination with ionizing radiation has been shown to induce autophagy in PC-3 cells by inhibition of Akt/mTORC1 signaling [212]. In contrast, milk-stimulated anabolic and pro-survival mTORC1 signaling impairs the appropiate mTORC1 balance for maintaining sufficient autophagy and may attenuate autophagy-inducing therapeutic strategies to kill tumor cells. A persistent impairment of the appropriate autophagy balance by continued cow´s milk-activated mTORC1 signaling may thus contribute to the initiation of tumorigenesis because reduced autophagy leads to deficient clearance of cells with acquired DNA damage and genetic instability [200]. In this regard, persistent cow´s milk consumption with accelerated mTORC1 signaling may not only contribute to the progression of already established tumor cells but may promote the initiation steps of prostate tumorigenesis.

### Glucocorticoids inhibit mTORC1 signaling and promote autophagy and tissue atrophy

It has been demonstrated that dexamethasone treatment decreased epithelial cell proliferation of the rat ventral prostate and reduced mTOR signaling [213]. Recently, the molecular crosstalk between glucocorticoid receptor (GR) and mTORC1 signaling has been elaborated [214]. A well known adverse effect of prolonged systemic glucocorticoid treatment is tissue atrophy, the result of increased cellular autophagy. In skeletal muscle, direct target genes of GR signaling involve the protein REDD1 (regulated in development and DNA damage responses) and the transcription factor KLF15 (Krüppel-like factor-15). Both inhibit mTORC1 activity, although via distinct mechanisms. The REDD1 gene is activated at the promoter level by ligand-bound GR and is transcriptionally induced under stress conditions like hypoxia (via HIF1α), which appears necessary for the downregulation of mTORC1 signaling during stress conditions [215]. REDD1 functions upstream of TSC2 and Rheb in order to downregulate mTORC1 signaling in response to glucocorticosteroids [215-217].

KLF15 upregulates gene expression of BCAT2, a mitochondrial enzyme, catalyzing the first step in the catabolism of BCAAs to accelerate BCAA degradation [218]. The glucocorticoid-driven GR-KLF15-BCAT2 axis may negatively modulate the intracellular availability of BCAAs resulting in a negative impact on mTORC1 function in skeletal muscle. Glucocorticoid-mediated downregulation of mTORC1 is not only a superb explanation for glucocorticoid-induced muscle atrophy, but also for the observed inhibitory effects on epithelial cell proliferation of the prostate of glucocorticoid-treated rats [213].

### Metformin antagonizes leucine-mediated mTORC1 signaling

Recent evidence points to cancer preventive and antineoplastic activities of the anti-diabetic drug *metformin* in PCa [219]. Currie *et al.*[220] reported that PCa mortality, which is increased in patients with type 2 diabetes, was reduced by metformin monotherapy in comparison to monotherapy with sulfonylureas or insulin. Metformin has been associated with improved overall survival of diabetic PCa patients [221]. Available data support the potential dual benefit of metformin on ADT-induced metabolic syndrome and its antineoplastic activity in PCa [222]. Metformin induced an up to 50% decrease in cell viability in human PCa cell lines (DU145, PC-3, and LNCaP) compared with only a modest effect (20% decrease) in P69 cells, a normal prostate epithelial cell line, indicating that metformin may specifically target the proliferation of PCa cells over normal cells [222]. Metformin-induced cell cycle inhibition was accompanied by a strong decrease of the level of cyclin D_1_ and an increase in p27 protein expression, which is typically observed by inhibition of mTORC1 [46,96,222]. In contrast, AR stimulation by DHT in LNCaP cells caused increased expression of cyclin D_1_, D_2_ and D_3_ and stimulated cell cycle progression [96]. Metformin inhibits mitochondrial respiratory chain complex I, which reduces ATP production, and thereby activates LKB1 and AMPK [223]. AMPK inhibits mTORC1 by direct phosphorylation of TSC2 and Raptor [57,60,224] (Figure 5). It has been demonstrated that metformin in combination with the glucose antagonist 2-deoxyglucose induced AMPK-dependent apoptosis of PCa cells [225].

Recently, a further inhibitory mode of metformin action on mTORC1 activity has been demonstrated, which interferes with leucine signaling. Metformin and phenformin have been shown to inhibit amino acid-dependent Rag GTPase-mediated mTORC1 activation [226]. Metformin disturbed leucine-dependent Rag GTPases required for translocation of inactive mTORC1 to activated Rheb enriched in lysosomal membranes, thereby reducing mTORC1 activity [53,55,227]. Similar to the effect of amino acid withdrawal, treatment of cells growing in amino acid-rich media in the presence of phenformin caused mTORC1 to leave the perinuclear intracellular compartment and to disperse throughout the cytoplasm without affecting amino acid steady state levels. It is thus conceivable that the biguanide metformin (C_4_H_11_N_5_; molar mass 129.1) functions as a competitive inhibitor of leucine (C_6_H_13_NO_2_; molar mass 131.2) in the Rag GTPase-dependent process of mTORC1 activation. Notably, the daily dose of metformin (2 g/day) is in the range of leucine intake derived from daily consumption of 100 g meat or cheese. Thus, disruption of leucine-mediated mTORC1-signaling by metformin may explain metformin´s antineoplastic activity in PCa. Moreover, the combination of metformin treatment of patients receiving ADT would potentiate the treatment effect on mTORC1 signaling as ADT reduces intracellular leucine uptake, while metformin suppresses leucine-mediated translocation of inactive mTORC1 to lysosomal compartments enriched in activated Rheb.

### Decreased leucine uptake and attenuated mTORC1 signaling by plant-based diets

The balance of a plant-based as opposed to dairy and animal protein-enriched diet has been appreciated as an important factor for the prevention of PCa [228]. Evidence has been provided that a diet emphasizing plant over animal product intake has been associated with decreased risk and less aggressive course for PCa [229]. On the other hand, the intake of dairy products and red meat has been associated with increased risk, while the intake of vegetables, especially cruciferous vegetables and tomato products, was associated with a decreased risk of PCa. [230-233]. The potential PCa-protective effect of a plant-based diet may be explained by the reduction of dairy- and animal meat-derived leucine intake, and especially lower insulin and IGF-1 signaling of non-dairy plant-based diets attenuating overall mTORC1 activity. Moreover, diets emphasizing plants, especially cruciferous vegetables, not only decrease leucine-dependent mTORC1 activation but they provide natural plant-derived inhibitors of mTORC1.

### Natural plant-derived mTORC1 inhibitors

mTORC1 activation by leucine-rich dairy consumption may be attenuated by natural plant-derived inhibitors of mTORC1. Increasing studies have demonstrated that *3,3´-diindolylmethane* (DIM), *epigallocatechin gallate* (EGCG), *genistein*, *curcumin*, *resveratrol* and *caffeine*, all inhibit mTORC1 signaling directly or indirectly and have been suggested to reduce the risk of PCa and other common cancers [234-251].

Especially, the consumption of cruciferous vegetables has been associated with a decreased risk of PCa [244,245,250]. 3,3´-Diindolylmethane (DIM) is generated in the acidic environment of the stomach following dimerization of indole-3-carbinol monomers present in cruciferous vegetables such as broccoli, brussel sprouts, cauliflower and cabbage. DIM suppresses signaling through Akt/mTORC1 pathways resulting in cell cycle arrest [250]. Recent studies have shown that PDGF-D and its cognate receptor PDGFR-β are expressed in prostate tumor tissues, suggesting that PDGF-D might play an important role in the development and progression of PCa. Overexpression of PDGF-D in PC-3 cells resulted in rapid growth and enhanced cell invasion that was associated with activation of mTORC1 [245]. B-DIM significantly inhibited both mTORC1 and Akt in PC-3 PDGF-D cells associated with decreased cell proliferation and less invasion [245].

Laboratory and clinical studies have demonstrated that green tea components, specifically the green tea catechin epigallocatechin gallate (EGCG), can induce apoptosis, suppress progression, and inhibit invasion and metastasis of PCa [251]. There is substantial animal and *in vitro* evidence supporting the chemopreventive effects of green tea polyphenols in PCa [252,253]. Tea polyphenols have been detected in prostate tissue in humans after green tea consumption [254]. Intriguingly, EGCG has been proven to function as an ATP-competitive inhibitor of both PI3K and mTORC1 catalytic units [241].

In LNCaP prostate cancer cells, the naturally occurring phytopolyphenol compound resveratrol inhibited the phosphorylation of PI3K, Akt and mTORC1 and induced growth arrest and apoptosis through mTORC1 inhibition and activation of FoxO transcription factors [255].

Thus, accumulating evidence from studies of plant-derived mTORC1 inhibitors implies that a higher consumption of vegetables, predominantly cruciferous vegetables in association with green tea consumption may antagonize increased mTORC1 signaling induced by high consumption of dairy proteins. Diets emphasizing vegetables and fruits and restricting dairy products, isolated sugars and cereal grains (which typically have high glycemic loads), like *Paleolithic type diets*, are associated with reduced insulin and IGF-1-signaling and higher intake of plant-derived mTORC1 inhibitors. It is likely that plants have evolved various natural mTORC1 inhibitors to defend invading opportunistic organisms by attenuating their TOR-dependent growth and metabolic activity.

### Dairy protein consumption during critical growth phases of the prostate gland

At least three major mTORC1-dependent growth phases exist in humans, which affect the growth and development of the prostate gland as well: fetal and postnatal growth of the prostate and sexual maturation of the prostate during puberty. The *Danish National Birth Cohort* from 50,117 mother-infant pairs provided evidence that increased total daily dairy protein consumption during pregnancy increased the neonate´s size at birth and was linearly correlated with increased birth weight [256]. Dairy protein-activated mTORC1 signaling may increase placental growth, which augments the transfer of glucose and amino acids to the fetus thus promoting fetal growth [257,258]. It is conceivable that increased dairy protein intake during pregnancy not only stimulates general growth and size of the fetus but may also affect the growth of the prostate during fetal development.

Prostatic branching morphogenesis is an intricate event requiring precise temporal and spatial integration of numerous hormonal and growth factor-regulated inputs. Gosh *et al.* recently demonstrated that PI3K/mTOR activity is robustly induced by androgen during murine prostatic development and showed that PI3K/mTOR signaling is necessary for prostatic epithelial bud invasion of surrounding mesenchyme [259]. These data point to an important role for PI3K/mTOR signaling in prostatic epithelial invasion and migration and implicate the maintenance of a fine tuned balance of PI3K-mTOR activity as a most critical regulatory circuit for prostatic epithelial morphogenesis [259]. It has been suggested that some of the same signaling pathways may be required for both prostatic morphogenesis and prostate tumorigenesis. Organ morphogenesis is not completed after birth and final organ maturation exceeds into the postnatal feeding period.

It is thus of serious concern that currently available cow´s milk-based infant formula provides excessive leucine per feeding content in comparison to the physiological leucine content of human breast milk [100-113]. Serum leucine, total IGF-1 as well as insulin serum concentrations are significantly higher in formula-fed infants compared to breast-fed infants [113]. Median serum concentrations of leucine at 6 months of age were lowest in breast-fed infants (106 μmol/L) compared to infants either fed low-protein formula (120 μmol/L) or high protein formula (165 μmol/L) [113]. Thus, cow´s milk-derived infant formula does not meet the physiological lower leucine signaling axis required for adequate mTORC1 regulation of the human newborn. These postnatal aberrations of leucine-mediated mTORC1 signaling may have an adverse effect on mTORC1-mediated postnatal prostate morphogenesis, thereby priming metabolic deviations that ultimately promote prostate tumorigenesis.

Not only the perinatal growth phase but also puberty-dependent growth and sexual maturation of the prostate requires appropriate signaling to maintain adequate mTORC1-activity during this sensitive period of sexual gland differentiation. In fact, recent epidemiological evidence supports the view that high intake of milk during puberty increased the risk of advanced PCa in adult life, thus questioning the attitude of promoting school milk consumption during puberty and adolescence [30].

### Targeted therapy for prostate cancer by inhibition of the PI3K/Akt/mTOR pathway

Leucine stimulation alone is sufficient to stimulate mTORC1 signal transduction [54]. Activation of mTORC1/S6K1 signaling is widespread in a number of human cancers [260]. Currently, oncology pays special attention to the prominent role of the activated PI3K/Akt/mTOR pathway in PCa, which has led to the development of multiple new drugs for targeted therapy of PCa [261-264]. Molecular changes in the PI3K/Akt/mTOR signaling pathway have been demonstrated to differentiate benign from malignant prostatic epithelium and are associated with increasing tumor stage, grade, and risk of recurrence [261-264]. AR transcriptional activity as well as AR expression is regulated by Akt [265]. In addition, androgens regulate the Akt pathway by both genomic and non-genomic effects [265]. At this stage, several inhibitors of the mTOR pathway are being assessed in laboratory and clinical trials underlining the pivotal role of aberrant mTOR signaling in PCa tumorigenesis [263]. It has recently been demonstrated that the ATP site mTOR inhibitor INK128 inhibited PCa progression in mice by inhibiting oncogenic mTORC1 signaling [91].

Pharmacological mTORC1 inhibitors thus counteract BCAA, insulin and IGF-1-induced overstimulation of mTORC1 signaling stimulated by the consumption of dairy protein-enriched Western diet.

## Conclusion

Routine cow´s milk consumption of another species` milk is an evolutionary novel dietary behavior that has the potential to alter human life history parameters and may have long-term adverse health effects [109]. Historically, the advantage of cow´s milk consumption has been associated with reduced infant mortality, improved fertility, increased BMI in children, earlier onset of menarche and increased linear growth during adolescence [105,106,109]. However, these at first glance “beneficial” effects may become adverse effects later in life, especially in Western populations with a higher life expectancy. The clinical manifestation of PCa is a disease of the elderly. However, the initiation phase of PCa may begin as early as fetal growth.

Our understanding of mammalian milk has changed from a “simple food” to a species-specific endocrine signaling system. Milk´s functionality depends on its stimulation of the nutrient-sensitive kinase mTORC1, the critical hub regulating cell growth, proliferation, autophagy and metabolic programming. Increased cow´s milk and dairy protein consumption during pregnancy, the period of postnatal infant formula feeding and milk/dairy protein consumption in childhood, adolescence as well as adulthood may promote and maintain abnormally high mTORC1 signaling modifying physiological signaling and mRNA patterns required for adequate tissue morphogenesis and programming resulting in long-term adverse effects on prostate health. Dairy protein-derived activation of mTORC1 signaling may disturb most sensitive “windows” of mTORC1-dependent metabolic programming and autophagy regulation thereby upsetting developmental programming and regular differentiation steps of the prostate gland (Figure 4B). This line of thought may explain the observed cancer-protective effect of prolonged breast-feeding, which ensures the physiological mTORC1-signaling axis provided by human milk. Increased mTORC1-signaling due to cow´s milk consumption during puberty may explain the association between frequent cow´s milk consumption during adolescence and higher risk of aggressive PCa in adulthood [30,266]. 

Sufficient evidence has accumulated to justify the rejection of the “high dairy calcium –low vitamin D-hypothesis” of PCa [38], whereas the *intrinsic signaling capability of milk proteins* along with orally bioavailable *estrogen metabolites* present in milk from pregnant cows represents a more likely explanation for the role of dairy consumption in the development and progression of PCa in Western countries [6,10,42]. As mTORC1-mediated cow´s milk signaling shares the same downstream pathways as oncogenic mTORC1 signaling of prostate epithelial cells with growth-promoting genetic aberrations, increased intake of cow´s milk and dairy proteins may exaggerate already increased mTORC1 signaling of PCa cells. Thus, cow´s milk is not a simple nutrient but an endocrinological effector that provides a pro-survival and anti-autophagy tissue environment promoting the initiation and progression of PCa (Figure 5). In contrast, plant-based dietary regimens are associated with reduced intake of insulinotropic and IGF-1 elevating amino acids as well as cow´s milk-derived estrogens and in contrast increase the uptake of potential cancer-preventive natural mTORC1 inhibitors. Hormonal ablation therapy (ADT), metformin treatment, natural mTORC1 inhibitors and targeted therapy with synthetic mTORC1 inhibitors just share a common mode of action: the attenuation of increased mTORC1 activity, which is increased by high milk and dairy protein consumption.

In 1994, Ross and Henderson [267] have asked: *“Do diet and androgens alter prostate cancer risk* via *a common etiologic pathway?”* Today, this question may be well explained by androgen- and milk-mediated synergisms in prostate mTORC1 signaling [97]. From an evolutionary perspective it can be concluded that the persistent “abuse” of the growth-promoting signaling system of bovine milk by humans over their entire life span maintains the most important hallmark of cancer biology, i.e., sustained proliferative signaling [102]. The magnitude of mTORC1 signaling of Western diet appears to be much higher than that of Paleolithic diets [133], which are less insulinotropic, provide lower glycemic load and exclude the intake of cow´s milk proteins [268-271]. In the 1930s leaders of pediatrics were convinced that human breast milk is “just food” [272]. According to this simplified misconception of milk´s biology no further efforts were undertaken to explore milk´s growth-promoting signal transduction pathways, allowing the widespread introduction of cow´s milk-based infant formula feeding. Subsequent evidence has clearly demonstrated that cow´s milk-based infant formula substantially exceeds the insulin, IGF-1 and leucine serum concentrations of formula-fed infants in comparison to breast-fed infants [110-113]. Daily intakes of 500 ml cow´s milk for children are still routinely recommended by leading pediatricians [158]. This advice means that one third of daily total protein intake (recommendation 56 g) in the age-range for 14-18 year-old boys will be administered in form of growth-promoting *signaling proteins* and not by *structural proteins* like meat or fish. Because adolescence represents a most critical mTORC1-dependent period of prostate differentiation and the subsequent increased risk for PCa [30], the medical community urgently needs to re-evaluate dietary milk recommendations. Dietary recommendations for children and adolescents require nutrient intakes, which allow an adequate and undisturbed period for prostate morphogenesis and sexual maturation of the prostate gland. In this context, it is of serious concern, that increasing numbers of adolescents and young men consume 60 to 80 g of highly insulinotropic whey protein concentrates daily to gain muscle mass [273]. Thus, future dietary studies should clarify the impact of increased cow´s milk protein intake upon early steps of prostate morphogenesis and differentiation. It will become important in future experimental work to define safe upper limits for long-term milk and dairy intake for the prevention of the most common dairy-promoted cancer in men.

## Abbreviations

ADT: Androgen deprivation therapy; Akt: Akt kinase (protein kinase B, PKB); AMP: Adenosine monophosphate; AMPK: AMP-activated protein kinase; AMP: Adenosine monophosphate; AR: Androgen receptor; ATF4: Activating transcription factor 4; ATP: Adenosine triphosphate; BCAAs: Branched-chain essential amino acids (leucine, isoleucine, valine); BCAT2: Branched-chain aminotransferase 2; 1,25(OH)_2_D: 1,25-Dihydroxyvitamin D; DIM: 3,3´-Diindolylmethane; DMBA: Dimethylbenz(a)anthracene; DHT: Dihydro-testosterone; ERK: Extracellular regulated MAP kinase; 4EBP1: Eukaryotic initiation factor (eIF) 4E-binding protein 1; EGCG: Epigallocatechin gallate; ER: Estrogen receptor; FoxO: Forkhead box class O transcription factor; GAP: GTPase activating protein; GDT: Guanosine diphosphate; GIP: Glucose-dependent insulinotropic polypeptide; GLUT: Glucose transporter protein; GTP: Guanosine triphosphate; IGF-1: Insulin-like growth factor 1; IGF1R: IGF-1 receptor; IR: Insulin receptor; IRS-1: Insulin receptor substrate 1; LAT: L-type amino acid transporter; LKB1: Liver kinase B1; MEK: Mitogen-activated protein kinase; mTORC1: Mammalian target of rapamycin complex 1; mTORC2: Mammalian target of rapamycin complex 2; PCa: Prostate cancer; PDGF: Platelet-derived growth factor; PDGFR: Platelet-derived growth factor receptor; PI3K: Phosphoinositol-3 kinase; PDK-1: Phosphoinositide-dependent kinase 1; PPARγ: Peroxisome proliferator-activated receptor gamma; PRAS40: Proline-rich Akt substrate 40; PTEN: Phosphatase and tensin homologue deleted on chromosome 10; Raptor: Regulatory-associated protein of mTOR; Ras: Rat Sarcoma virus oncogene; Rictor: Rapamycin-insensitive companion of mTOR; Rheb: Ras homolog enriched in brain; RSK: P90 ribosomal S6 kinase; S6K1: P70 S6 kinase 1; SREBP-1: Sterol regulatory element-binding protein-1; TSC1: Tuberous sclerosis complex 1 (hamartin); TSC2: Tuberous sclerosis complex 2 (tuberin).

## Competing interests

BC Melnik, S M John, P Carrera-Bastos, and L Cordain declare that they have no competing interests.

## Authors`contributions

BCM was responsible for all translational research, data collection and characterization of the signaling pathways of milk proteins and prostate cancer cells. SMJ, PCB, and LC validated all nutrition-related publications on milk and dairy consumption and their validity in relation to increased risk of prostate cancer. BCM has primary responsibility for the final content. All authors revised, discussed and approved the final manuscript.
